# Prevalence of economic burden of overweight and obesity and their associated factors in Saudi Arabia

**DOI:** 10.2478/abm-2026-0013

**Published:** 2026-04-30

**Authors:** Mubashir Zafar, Abdullah Ali Albrahimi, Naif Mohammed Salem Alanezi

**Affiliations:** 1 Family and Community Medicine Department, College of Medicine, University of Hail, Hail 2440, Saudi Arabia ubashizafar6@gmail.com; 2 College of Medicine, University of Hail, Hail 2440, Saudi Arabia

**Keywords:** body mass index, cost-of-illness study, economic burden, healthcare costs, obesity, overweight

## Abstract

**Background:**

Overweight and obesity represent significant public health challenges in Saudi Arabia with substantial economic implications.

**Objective:**

To estimate the economic burden of overweight and obesity in Saudi Arabia and identify associated factors.

**Methods:**

This cross-sectional study utilized a mixed-methods approach combining primary survey data with secondary national health data. A total of 667 participants were recruited through stratified random sampling using a validated structured questionnaire. Direct medical costs were estimated using healthcare utilization data, while indirect costs were calculated using the human capital approach. Logistic regression analysis identified factors associated with high economic burden. Statistical significance was set at *P* < 0.05.

**Results:**

The total annual economic burden of overweight and obesity in Saudi Arabia was estimated at SAR 7.6 billion (US$2.0 billion), representing 1.2% of GDP. Direct medical costs accounted for SAR 5.2 billion, while indirect costs totaled SAR 2.4 billion. Among participants, 46.8% experienced a high individual economic burden. Younger age (18–30 years) was significantly associated with higher economic burden compared to older adults (adjusted odds ratio [AOR] = 2.9, 95% confidence interval [CI]: 2.2–4.0, *P* = 0.004). Primary education level was associated with higher economic burden compared to university education (AOR = 3.9, 95% CI: 2.0–9.2, *P* = 0.045). Body mass index (BMI) ≥ 30 kg/m^2^ was associated with higher economic burden (AOR= 3.0, 95% CI: 2.2–3.7, *P* = 0.001).

**Conclusion:**

The economic burden of overweight and obesity in Saudi Arabia is substantial and disproportionately affects younger adults, individuals with lower education, and those with higher BMI. These findings support targeted interventions and policy initiatives aligned with Saudi Vision 2030 healthcare transformation goals.

Overweight and obesity have emerged as major public health challenges in Saudi Arabia, with prevalence rates reaching 35.5% for obesity among adults, representing one of the highest rates in the Middle East [1]. This epidemiological shift has created substantial economic implications requiring comprehensive assessment to inform healthcare policy and resource allocation.

The economic impact of obesity extends beyond direct healthcare expenditures to include significant indirect costs through productivity losses and premature mortality [2]. While global studies have documented substantial economic burdens, comprehensive cost-of-illness analyzes specific to Saudi Arabia remain limited, particularly those incorporating recent postpandemic data and both direct and indirect cost components [3].

Contributing factors to rising obesity prevalence in Saudi Arabia include lifestyle changes, dietary transitions toward processed foods, reduced physical activity, and rapid urbanization [4]. Socioeconomic determinants such as education, income, and occupation significantly influence both obesity prevalence and associated economic burden [5].

This study’s unique contribution lies in providing a comprehensive cost-of-illness analysis using recent data to estimate both direct medical costs and indirect productivity costs, while examining individual-level factors associated with high economic burden. These findings directly support Saudi Vision 2030’s emphasis on healthcare system transformation and economic sustainability through evidence-based planning [6].

This cost-of-illness study aims to estimate the total economic burden of overweight and obesity in Saudi Arabia, quantify direct and indirect cost components, and identify sociodemographic factors associated with individual-level economic burden.

## Materials and methods

### Study design and population

This cross-sectional cost-of-illness study was conducted between January and August 2024, employing a mixed-methods approach combining primary data collection with secondary data analysis from national health databases and economic reports. The target population included Saudi adults aged 18 years and above across multiple regions. The protocol has been approved by the research ethics committee. All procedures involving human participants complied with the 1964 Helsinki Declaration.

### Sample size and sampling method

Sample size calculation was based on an estimated obesity prevalence of 35.5%, margin of error of 4%, and 95% confidence level, yielding a minimum required sample of 667 participants. To ensure adequate power for subgroup analyses and account for potential non-response, 667 participants were successfully recruited using stratified random sampling to ensure representation across regions, urban/rural areas, and socioeconomic strata.

### Data collection instruments

#### Questionnaire development and validation

A structured questionnaire was developed based on established instruments and validated through a 3-stage process: (1) content validity assessment by 5subject matter experts, (2) pilot testing with 50 participants, and (3) test-retest reliability assessment (κ = 0.84). The questionnaire captured sociodemographic information, healthcare utilization patterns, work productivity measures, and self-reported health status.

#### Anthropometric measurements

Trained data collectors measured height and weight using standardized protocols and calibrated equipment. Body mass index (BMI) was calculated as weight (kg) divided by height (m^2^), with obesity defined as BMI ≥ 30 kg/m^2^ and overweight as BMI 25-29.9 kg/m^2^ according to WHO criteria.

### Economic burden assessment

#### Direct medical costs

Direct costs included healthcare expenditures for obesity-related conditions (diabetes, hypertension, cardiovascular disease, sleep apnea, musculoskeletal disorders). Cost estimates were obtained from national insurance reimbursement data, adjusted to 2024 values using healthcare-specific inflation rates.

#### Indirect cost calculation methods

Indirect costs were calculated using the human capital approach with the following components:

*Productivity losses (Absenteeism):* Based on participant-reported sick leave days multiplied by occupation-specific average daily wages.

*Reduced productivity (Presenteeism):* Estimated using the work productivity and activity impairment (WPAI) questionnaire, with productivity reduction percentages applied to daily wage rates.

*Premature mortality*: Years of potential life lost calculated using life tables and age-gender-specific mortality rates for obesity-related conditions, multiplied by projected lifetime earnings based on education and occupation categories.

#### Outcome variable definition

Individual economic burden was dichotomized as “high” (annual costs ≥ SAR 15,000) or “low” (< SAR 15,000) based on the 75th percentile of the total cost distribution, incorporating both direct medical costs and estimated productivity losses for each participant.

#### Statistical analysis

Descriptive statistics summarized participant characteristics and cost distributions. Chi-square tests examined bivariate associations. Multivariate logistic regression identified factors associated with high economic burden, with model building using forward selection (*P* < 0.10 for entry, *P* < 0.05 for retention). Variance inflation factors (VIF) assessed multicollinearity, with VIF > 5 indicating concern.

#### Accuracy and reliability checks

Data entry was double verified for 20% of cases. Cost calculations were independently verified by 2 researchers. Sensitivity analyses used alternative cost estimation methods and burden thresholds (50th and 90th percentiles). Missing data (<5% for all variables) were handled using listwise deletion. Statistical significance was set at *P* < 0.05. All analyses used SPSS version 28.0.

## Results

### Participant characteristics

Among 667 participants, the mean age was 32.3 years (standard deviation [SD] ± 1.3), with 58.1% aged 18–30 years. Females comprised 68.7% of the sample, 76.9% had university education, and 52.1% were single. Regarding BMI, 73.0% were overweight, and 27.0% were obese (**Table 1**).

**Table 1.** Socio-demographic characteristics of the study participants (n = 667)

Variable	Number (%)
Gender	
Male	209 (31.3)
Female	458 (68.7)
Age (Mean 32.25, SD ± 1.28)	
18-30	387 (58.1)
31-60	280 (41.9)
Education status	
Primary	72 (10.8)
Secondary	82 (12.3)
University	513 (76.9)
Marital status	
Single	347 (52.1)
Married	320 (47.9)
Smoking	
Ever	596 (89.3)
Never	71 (10.7)
Occupation	
Student	27 (4.1)
Business	89 (13.3)
Job	551 (82.6)
Past medical or surgical history	
Yes	204 (30.6)
No	463 (69.4)
Body mass index (BMI)	
25-29.9	487 (73.0)
>30	180 (27.0)

### Economic burden estimates

#### National-level cost-of-illness analysis

The total annual economic burden of overweight and obesity in Saudi Arabia was estimated at SAR 7.6 billion (equivalent to US$2.03 billion using the 2024 exchange rate of 3.8 SAR/USD), representing approximately 1.2% of national GDP.

#### Direct medical costs: SAR 5.2 billion (68.4%)

Diabetes management: SAR 1.3 billion, cardiovascular diseases: SAR 1.5 billion, Hypertension management: SAR 1.0 billion, other obesity-related conditions: SAR 1.4 billion.

#### Indirect costs: SAR 2.4 billion (31.6%)

Productivity losses (absenteeism): SAR 1.4 billion, reduced productivity (presenteeism): SAR 0.7 billion, premature mortality: SAR 0.3 billion.

### Individual-level economic burden

Among participants, 312 (46.8%) were classified as having a high economic burden. Mean annual costs were SAR 18,750 (SD ± 12,400) for high burden and SAR 8,200 (SD ± 4,100) for low burden categories.

### Factors associated with high economic burden

Multivariate logistic regression analysis (**Table 2**) revealed several significant associations after adjusting for confounders:

Age 18–30 years vs. 31–60 years: adjusted odd ratio (AOR) = 2.9 (95% confidence interval [CI]: 2.2–4.0, *P* = 0.004), Primary education vs. university: AOR = 3.9 (95% CI: 2.0–9.2, *P* = 0.045), No past medical history: AOR = 3.8 (95% CI: 2.9–5.4, *P* < 0.001), BMI ≥ 30 vs. 25-29.9: AOR = 3.0 (95% CI: 2.2–3.7, *P* = 0.001).

**Table 2.** Association of economic burden of obesity with sociodemographic characteristics among study participants (n = 667)

Characteristics	High economic burden un-adjusted OR [95% CI] (*P* value)	High economic burden AOR [95% CI] (*P* value)
Age category (years)		
31-60	1	1
18-30	3.3 [2.5-4.4] (0.000)	2.9 [2.2-4.0] (0.004)
Gender		
Male	1	1
Female	2.5 [2.0-3.2] (0.075)	2.2 [1.7-2.8] (0.553)
Marital status		
Single	1	1
Married	3.5 [2.7-4.8] (0.000)	2.6 [1.8-3.6] (0.172)
Education status		
University	1	1
Secondary	3.6 [2.1-8.0] (0.023)	2.2 [1.1-3.8] (0.535)
Primary	1.5 [0.3-2.0] (0.046)	3.9 [2.0-9.2] (0.045)
Smoking status		
Ever	1	1
Never	2.9 [2.1-4.4] (0.023)	2.3 [1.6-3.6] (0.427)
Occupation		
Student	1	1
Business	1.7 [1.2-3.4] (0.588)	1.5 [1.0-2.7] (0.021)
Job	2.3 [1.4-5.0] (0.598)	2.0 [1.0-5.0] (0.983)
Past medical and surgical history		
Yes	1	1
No	4.2 [3.1-6.0] (0.000)	3.8 [2.9-5.4] (0.000)
BMI		
25-29.9	1	1
≥30	2.4 [1.9-3.1] (0.162)	3.0 [2.2-3.7] (0.996)

1 AOR, adjusted odd ratio; BMI, Body mass index; CI, confidence interval; OR, odd ratio.

### Sensitivity analysis results

Alternative cost calculation methods and burden thresholds yielded consistent findings, with effect sizes varying by <15%. No significant multicollinearity was detected (all VIF values <3.0).

## Discussion

This cost-of-illness study provides the first comprehensive assessment of obesity’s economic burden in Saudi Arabia using recent data and incorporating both direct medical costs and indirect productivity costs. The estimated annual burden of SAR 7.6 billion (1.2% of GDP) represents a substantial economic impact requiring urgent policy attention.

**Figure 1.**
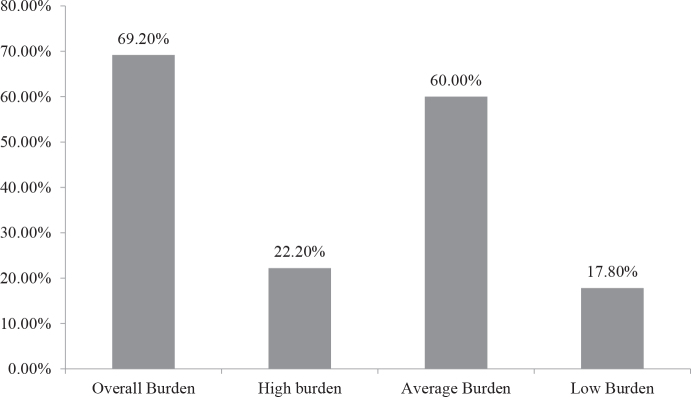
Categories represent self-reported perceived economic burden due to obesity.

**Figure 1** Our findings indicate a higher economic burden compared to earlier Saudi estimates, which primarily focused on direct costs and reported figures around 0.8%–1.0% of GDP [7]. This increase likely reflects both rising prevalence rates and more comprehensive cost accounting, including productivity losses. Internationally, our estimate aligns with similar middle-income countries, where obesity-related costs typically range from 1.0% to 3.0% of GDP [8–10].

**Figure 2.**
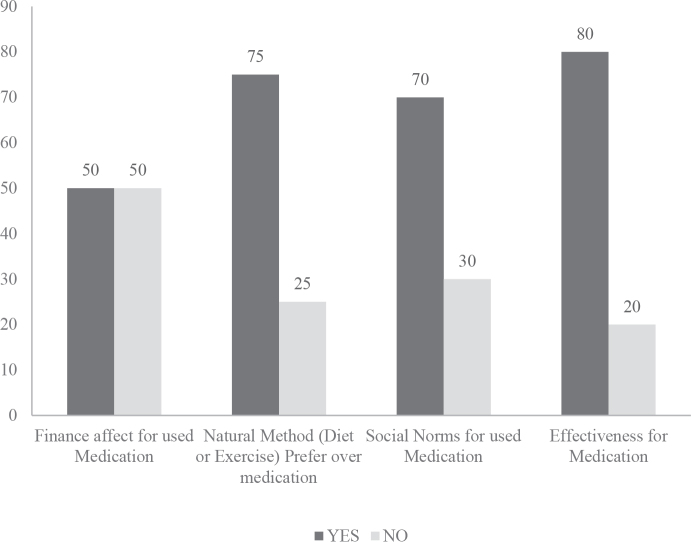
Factors influencing medication used for obesity.

**Figure 2** The direct cost proportion (68.4%) is consistent with cost-of-illness studies from comparable healthcare systems, though the substantial indirect costs (31.6%) underscore obesity’s broader economic impact beyond healthcare expenditure [11, 12].

The association between younger age (18–30 years) and higher individual economic burden contrasts with typical patterns where older adults bear greater healthcare costs. This finding may reflect higher healthcare utilization among younger adults with early onset obesity-related complications, or greater productivity losses in the economically active population. This pattern has significant implications for workforce productivity and long-term economic sustainability.

The strong association between lower education (primary level) and higher economic burden highlights important health equity considerations. Individuals with limited education face nearly 4 times higher odds of high economic burden, suggesting that educational interventions and targeted support programs should be prioritized in obesity prevention strategies.

These findings directly support Saudi Vision 2030’s healthcare transformation objectives by providing quantitative evidence for prioritizing obesity prevention and management. The substantial economic burden justifies investment in comprehensive prevention programs, particularly those targeting younger adults and individuals with lower socioeconomic status.

The finding that individuals without past medical history experience higher economic burden suggests opportunities for early intervention and preventive care strategies that could reduce long-term costs while improving health outcomes.

Direct medical costs of SAR 5.2 billion annually represent a significant strain on healthcare resources, with diabetes and cardiovascular disease management comprising over half of these costs. This distribution supports prioritizing integrated chronic disease management programs and preventive interventions targeting metabolic risk factors.

Several important limitations should be acknowledged. While stratified sampling was employed, the sample may not fully represent all Saudi regions, socioeconomic groups, or age distributions. The higher proportion of university-educated participants (76.9%) may limit generalizability to the broader population. Productivity measures, healthcare utilization, and some behavioral factors relied on self-report, potentially introducing recall bias and social desirability bias. Validation against objective measures was not possible for all variables. The study design precludes establishing causal relationships between identified factors and economic burden. Longitudinal studies are needed to understand temporal relationships and burden trajectories. Projecting national-level costs from a sample of 667 participants involves substantial uncertainty. While sensitivity analyses support our estimates, the extrapolation represents a significant methodological limitation requiring cautious interpretation. Combining multiple data sources (survey data, national databases, and economic reports) may introduce inconsistencies or double-counting despite efforts to ensure accuracy. The human capital method for indirect costs may not capture all productivity impacts and assumes full employment, potentially over- or under-estimating true economic losses.

Longitudinal studies tracking economic burden over time would provide valuable insights into cost trajectories and intervention effectiveness. Regional analyses could identify geographic variations in burden and inform targeted resource allocation. Economic evaluation of specific prevention and treatment interventions would support evidence-based policy decisions.

## Conclusion

The economic burden of overweight and obesity in Saudi Arabia is substantial, with annual costs of SAR 7.6 billion representing 1.2% of GDP. This burden disproportionately affects younger adults, individuals with lower education levels, and those with higher BMI categories. These findings provide strong economic justification for comprehensive obesity prevention and management programs aligned with Saudi Vision 2030 objectives. Priority should be given to interventions targeting health equity, particularly for younger adults and individuals with lower socioeconomic status. Longitudinal studies are needed to establish causal relationships and evaluate intervention effectiveness. Economic evaluation of specific prevention strategies would inform resource allocation decisions. The substantial economic burden supports investment in integrated chronic disease prevention and management programs, with emphasis on early intervention and accessible evidence-based treatments.
